# Crystal structures of 4-meth­oxy-*N*-(4-methyl­phenyl)benzene­sulfonamide and *N*-(4-fluoro­phenyl)-4-meth­oxy­benzene­sulfonamide

**DOI:** 10.1107/S2056989015019787

**Published:** 2015-10-28

**Authors:** Vinola Z. Rodrigues, C. P. Preema, S. Naveen, N. K. Lokanath, P. A. Suchetan

**Affiliations:** a Department of PG Studies and Research in Chemistry, St Aloysius College, Mangalore, India; b Institution of Excellence, University of Mysore, Mysuru-6, India; c Department of Physics, University of Mysore, Mysuru-6, India; d Department of Chemistry, University College of Science, Tumkur University, Tumkur 572 103, India

**Keywords:** crystal structure, *N*-(ar­yl)aryl­sulfonamides, C—H⋯O inter­actions, C—H⋯π inter­actions

## Abstract

Crystal structures of two *N*-(ar­yl)aryl­sulfonamides, namely, 4-meth­oxy-*N*-(4-methyl­phen­yl)benzene­sulfonamide, C_14_H_15_NO_3_S, (I), and *N*-(4-fluoro­phen­yl)-4-meth­oxy­benzene­sulfonamide, C_13_H_12_FNO_3_S, (II), were determined and analyzed. In (I), the benzene­sulfonamide ring is disordered over two orientations, in a 0.516 (7):0.484 (7) ratio, which are inclined to each other at 28.0 (1)°. In (I), the major component of the sulfonyl benzene ring and the aniline ring form a dihedral angle of 63.36 (19)°, while in (II), the planes of the two benzene rings form a dihedral angle of 44.26 (13)°. In the crystal structure of (I), N—H⋯O hydrogen bonds form infinite *C*(4) chains extended in [010], and inter­molecular C—H⋯π_ar­yl_ inter­actions link these chains into layers parallel to the *ab* plane. The crystal structure of (II) features N—H⋯O hydrogen bonds forming infinite one dimensional *C*(4) chains along [001]. Further, a pair of C—H⋯O inter­molecular inter­actions consolidate the crystal packing of (II) into a three-dimensional supra­molecular architecture.

## Abstract 2

In the crystal structures of 4-meth­oxy-*N*-(4-methyl­phen­yl)benzene­sulfonamide and *N*-(4-fluoro­phen­yl)-4-meth­oxy­benzene­sulfonamide, the supra­molecular architecture of the former is controlled by C—H⋯π_ar­yl_ inter­actions, forming a two-dimensional architecture, while in the latter, a pair of C—H⋯O inter­molecular inter­actions lead to the formation of a three-dimensional architecture.

## Chemical context   

Sulfonamide drugs were the first among the chemotherapeutic agents to be used for curing and preventing bacterial infection in human beings (Shiva Prasad *et al.*, 2011 ▸). They play a vital role as a key constituent in a number of biologically active mol­ecules. Up to now, sulfonamides have been known to exhibit a wide variety of biological activities, such as anti­bacterial (Subhakara Reddy *et al.*, 2012 ▸; Himel *et al.*, 1971 ▸), anti­fungal (Hanafy *et al.*, 2007 ▸), anti­inflamatory (Kuçukguzel *et al.*, 2013 ▸), anti­tumor (Ghorab *et al.*, 2011 ▸), anti­cancer (Mansour *et al.*, 2011 ▸), anti-HIV (Sahu *et al.*, 2007 ▸) and anti­tubercular activities (Vora & Mehta, 2012 ▸). In recent years, extensive research studies have been carried out on the synthesis and evaluation of pharmacological activities of mol­ecules containing the sulfonamide moiety for different activities, and have been reported to be important pharmacophores (Mohan *et al.*, 2013 ▸).

With these considerations in mind and based on our structural study of *N*-(4-substituted-phen­yl)-4-meth­oxy­benzene­sulfonamides (Vinola *et al.*, 2015 ▸), we report herein the crystal structures of 4-meth­oxy-*N*-(4-methyl­phen­yl)benzene­sulfonamide, (I), and *N*-(4-fluoro­phen­yl)-4-meth­oxy­benzene­sulfonamide, (II).

## Structural commentary   

**Figure 1:**
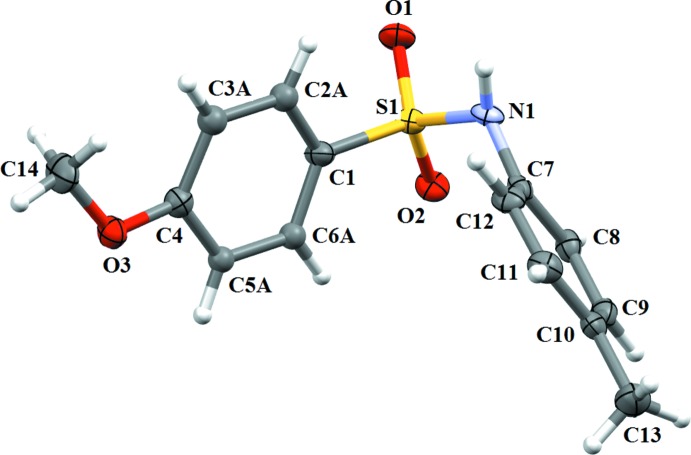
A view of (I), showing the atom labelling. Displacement ellipsoids are drawn at the 50% probability level. Only the major component of the disordered benzene ring is shown.

In (I) (Fig. 1 ▸), the benzene­sulfonamide ring is disordered due to rotation across the C_ar_—S(O_2_) bond over two orientations, with atoms C2, C3, C5 and C6 occupying two positions with a 0.516 (7):0.484 (7) ratio. The dihedral angle between the two parts of disordered benzene ring, *i.e.* C1/C2*A*/C3*A*/C4/C5*A*/C6*A* and C1/C2*B*/C3*B*/C4/C5*B*/C6*B*, is 28.0 (1)°. The dihedral angle between the sulfonyl benzene ring (considering the major component) and the aniline ring is 63.36 (19)°, and the N—C bond in the C—SO_2_—NH—C segment has a *gauche* torsion with respect to the S=O bonds. Further, the mol­ecule is twisted at the S—N bond, with a C1—S1—N1—C7 torsion angle of 66.33 (19)°. The meth­oxy group in the sulfonyl­benzene ring is in the same plane as that of the major component of the disordered sulfonyl­benzene ring, the torsion angle C5*A*—C4—O3—C14 being −176.2 (4)°, while it deviates slightly from planarity with respect to the minor component, the C5*B*—C4—O3—C14 torsion angle being 165.9 (4)°.

**Figure 2:**
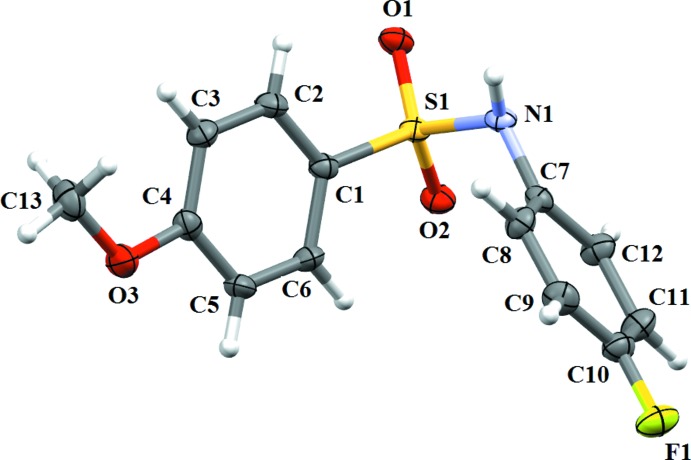
A view of (II), showing the atom labelling. Displacement ellipsoids are drawn at the 50% probability level.

In (II) (Fig. 2 ▸), the dihedral angle between the two benzene rings of 44.26 (13)° is less than that observed in (I), and the N—C bond in the C—SO_2_—NH—C segment has a *gauche* torsion with respect to the S=O bonds. Further, the mol­ecule is twisted at the S—N bond, with a C1—S1—N1—C7 torsion angle of 68.4 (2)°. Similar to (I), the meth­oxy group in the sulfonyl­benzene ring is in the same plane as that of the sulfonyl­benzene ring, the C5—C4—O3—C13 torsion angle being 177.0 (2)°.
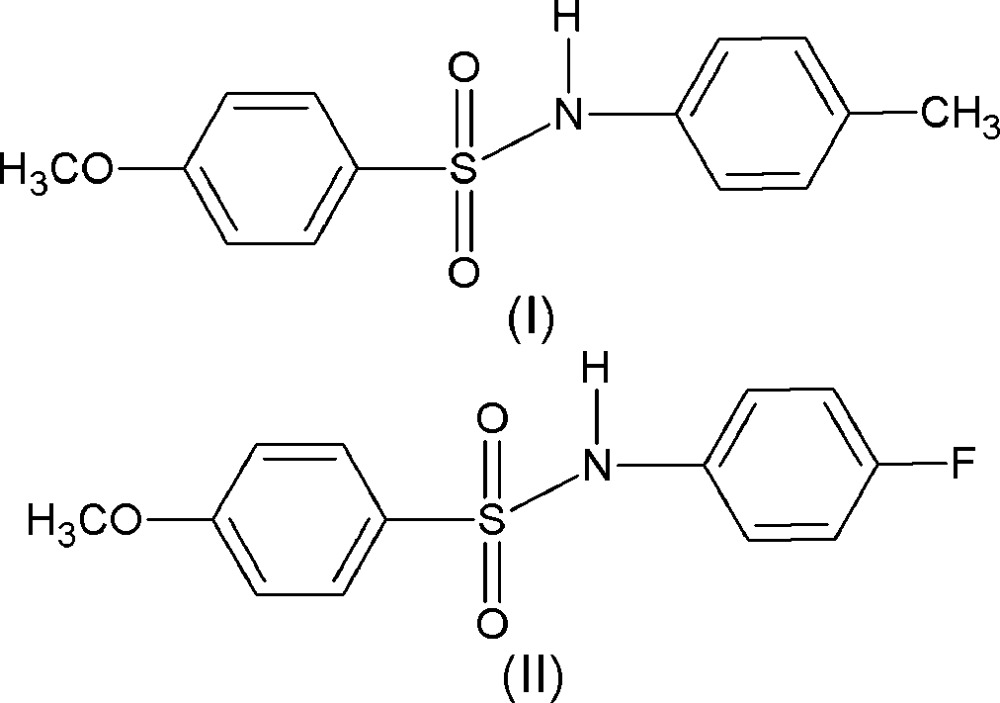


## Supra­molecular features   

**Figure 3:**
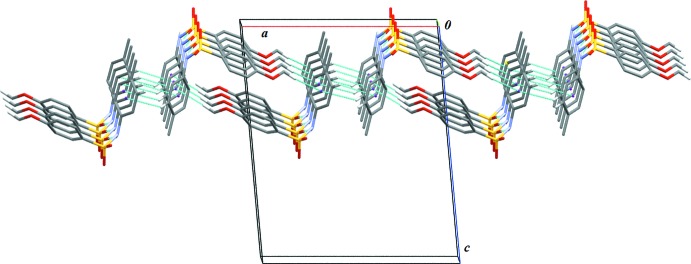
A portion of the crystal packing of (I), viewed approximately along [010] and showing inter­molecular hydrogen bonds as thin blue lines. Only the major component of the disordered benzene ring is shown. H atoms not involved in hydrogen bonding have been omitted for clarity.

In the crystal structure of (I), N1—H1⋯O2 hydrogen bonds (Table 1 ▸) link the mol­ecules into infinite one-dimensional *C*(4) chains along [010]. Neighbouring *C*(4) chains are inter­connected *via* C—H⋯π_ar­yl_ inter­actions (Table 1 ▸) into layers (Fig. 3 ▸) parallel to the *ab* plane.

**Figure 4:**
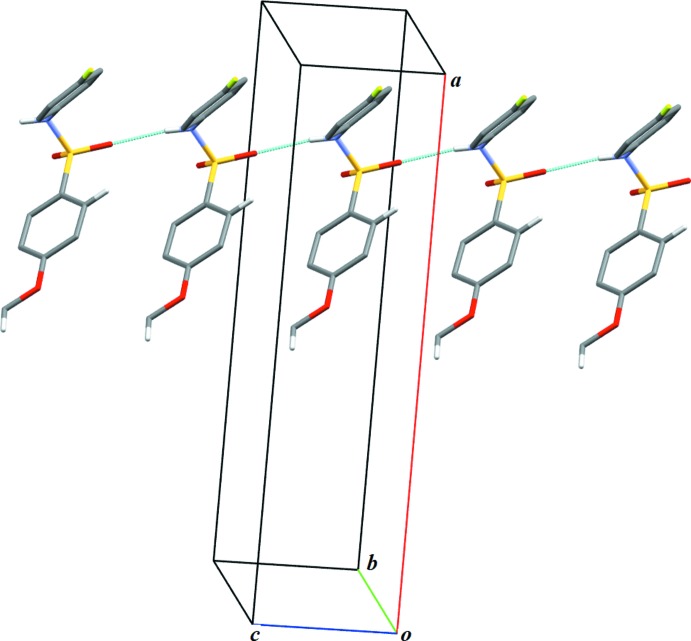
An N—H⋯O hydrogen-bonded (thin blue lines) chain of mol­ecules in the crystal structure of (II).

**Figure 5:**
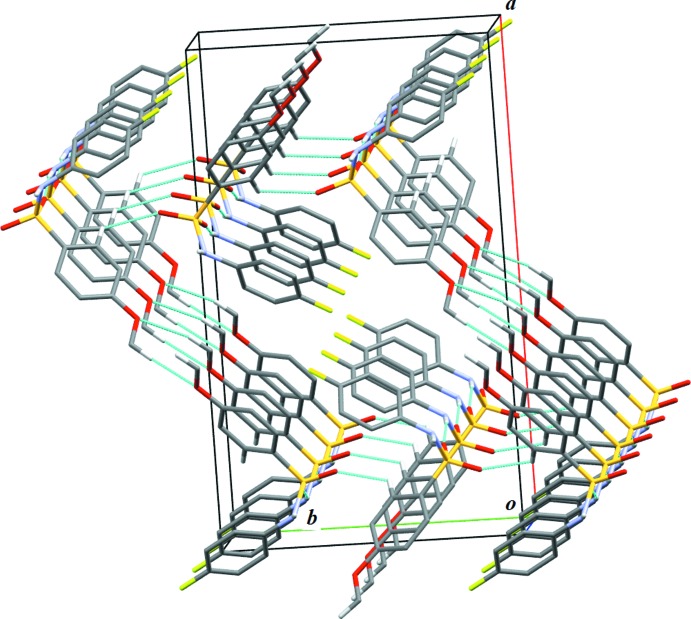
A portion of the crystal packing of (II). Thin blue lines denote inter­molecular C—H⋯O hydrogen bonds. H atoms not involved in hydrogen bonding have been omitted for clarity.

The crystal structure of (II) features N1—H1⋯O2 hydrogen bonds (Fig. 4 ▸ and Table 2 ▸), forming infinite one-dimensional *C*(4) chains along [001]. Further, weak inter­molecular C—H⋯O inter­actions (Table 2 ▸) consolidate the crystal packing of (II), leading to a three-dimensional supra­molecular architecture (Fig. 5 ▸).

## Database survey   

Three *N*-(4-substituted-phen­yl)-4-meth­oxy­benzene­sul­fon­am­ides (Vinola *et al.*, 2015 ▸), namely, 4-meth­oxy-*N*-(phen­yl)benzene­sulfonamide, (III), 4-meth­oxy-*N*-(4-meth­oxy­phen­yl)benzene­sulfonamide, (IV), and *N*-(4-chloro­phen­yl)-4-meth­oxy­benzene­sulfonamide, (V), have been reported previously. Compounds (IV) and (V) crystallize in monoclinic syngony, while compound (III) crystallizes in ortho­rhom­bic syngony. The dihedral angles between the two benzene rings in (III), (IV) and (V) are 55.1 (1), 56.3 (1) and 42.6 (1)°, respectively. Comparison of the dihedral angles between the two benzene rings in (I)–(V) shows that, when an electron-donating substituent is introduced into the *para* position of the aniline ring of (I) it results in a slight increase in the dihedral angle, whereas, when an electron-withdrawing substituent is introduced it decreases the dihedral angle. Further, the mol­ecules of (III), (IV) and (V) are twisted at the S—N bond, with C1—S1—N1—C7 torsion angles of −72.9 (1), 66.2 (1) and 72.5 (1)°, respectively. These values are similar to those observed in (I) and (II).

Comparison of the crystal structures (I) and (V) shows that the effect of introducing an electron-donating substituent into the *para* position of the aniline ring of (I) is quite different than that due to electron-withdrawing substituents. The crystal structure of (III) features N—H⋯O hydrogen bonds that form *C*(4) chains, and thus, the supra­molecular architecture is one-dimensional. In (IV), one N—H⋯O hydrogen bond and two alternating C—H⋯π_ar­yl_ (centroid of aniline ring) inter­actions direct a two-dimensional architecture. This is quite similar to the crystal structure of (I). Thus, the methyl and meth­oxy groups on the aniline ring have similar influence on the crystal structures of these compounds. However, the crystal structures of (II) and (V) are very different. The crystal structure of (V) features N—H⋯O hydrogen bonds that form *C*(4) chains. Further, (V) does not feature any structuredirecting inter­molecular inter­actions, and thus, the structure is one-dimensional. In contrast to this, the crystal structure of (II) features an N—H⋯O and two C—H⋯O inter­actions, leading to a three-dimensional architecture. Thus, the Cl and F atoms on the aniline ring have very different influences on the crystal structures of these compounds.

## Synthesis and crystallization   

Compounds (I) and (II) were prepared according to the literature method of Vinola *et al.* (2015 ▸). The purity of the compounds were checked by determining the melting points. Single crystals used for X-ray diffraction studies were obtained by slow evaporation of ethanol solutions of the compounds at room temperature.

## Refinement   

Crystal data, data collection and structure refinement details are summarized in Table 3 ▸. The amino H atoms were located in a difference map and were refined isotropically with the bond-length restraint N—H = 0.90 (1) Å. To improve considerably the values of *R*1, *wR*2, and *S* (goodness-of-fit), a partially obscured reflection (*i.e.* 100) was omitted from the final refinement of (I). The two parts (*A* and *B*) of the disordered benzene­sulfonyl ring in (I) were restrained to be planar (FLAT instruction), and thus, the r.m.s. deviations (considering non-H atoms) observed for the planes defining the two rings are 0.047 (1) (major-component ring *A*) and 0.054 (1) Å (minor-component ring *B*). The disordered atoms (C2, C3, C5 and C6) in both components were isotropically refined, and the C—C bond lengths were restrained to 1.391 (1) Å.

## Supplementary Material

Crystal structure: contains datablock(s) I, II, global. DOI: 10.1107/S2056989015019787/cv5497sup1.cif

Structure factors: contains datablock(s) I. DOI: 10.1107/S2056989015019787/cv5497Isup2.hkl

Supporting information file. DOI: 10.1107/S2056989015019787/cv5497Isup4.cml

Structure factors: contains datablock(s) II. DOI: 10.1107/S2056989015019787/cv5497IIsup3.hkl

Supporting information file. DOI: 10.1107/S2056989015019787/cv5497IIsup5.cml

CCDC reference: 1432501

Additional supporting information:  crystallographic information; 3D view; checkCIF report

## Figures and Tables

**Table 1:** Hydrogen-bond geometry (Å, °) for (I) *Cg* is the centroid of the C7–C12 ring.

*D*—H⋯*A*	*D*—H	H⋯*A*	*D*⋯*A*	*D*—H⋯*A*
N1—H1⋯O2^i^	0.89 (1)	2.13 (1)	3.010 (2)	170 (2)
C14—H14*B*⋯*Cg* ^ii^	0.96	2.70	3.541 (2)	146
C9—H9⋯*Cg* ^iii^	0.93	2.87	3.560 (2)	132

Symmetry codes: (i) 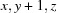; (ii) 
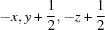
; (iii) 
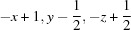
.

**Table 2:** Hydrogen-bond geometry (Å, °) for (II)

*D*—H⋯*A*	*D*—H	H⋯*A*	*D*⋯*A*	*D*—H⋯*A*
N1—H1⋯O2^i^	0.90 (1)	2.06 (1)	2.951 (3)	171 (3)
C6—H6⋯O1^ii^	0.93	2.55	3.192 (3)	127
C13—H13*B*⋯O3^iii^	0.96	2.60	3.468 (3)	151

Symmetry codes: (i) 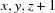; (ii) 
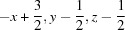
; (iii) 
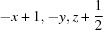
.

**Table 3:** Experimental details

	(I)	(II)
Crystal data		
Chemical formula	C_14_H_15_NO_3_S	C_13_H_12_FNO_3_S
*M*_r_	277.33	281.30
Crystal system, space group	Monoclinic, *P*2_1_/*c*	Orthorhombic, *P* *n* *a*2_1_
Temperature (K)	296	296
*a*, *b*, *c* (Å)	14.5604 (5), 5.2459 (2), 17.6094 (6)	20.2188 (13), 12.1199 (8), 5.1770 (3)
α, β, γ (°)	90, 95.205 (2), 90	90, 90, 90
*V* (Å^3^)	1339.50 (8)	1268.62 (14)
*Z*	4	4
Radiation type	Cu *K*α	Cu *K*α
μ (mm^−1^)	2.19	2.44
Crystal size (mm)	0.33 × 0.27 × 0.21	0.32 × 0.27 × 0.22

Data collection		
Diffractometer	Bruker APEXII	Bruker APEXII
Absorption correction	Multi-scan (*SADABS*; Bruker, 2009 ▸)	Multi-scan (*SADABS*; Bruker, 2009 ▸)
*T*_min_, *T*_max_	0.517, 0.632	0.481, 0.585
No. of measured, independent and observed [*I* > 2σ(*I*)] reflections	7071, 2139, 2047	5438, 1830, 1784
*R*_int_	0.042	0.037
(sin θ/λ)_max_ (Å^−1^)	0.583	0.583

Refinement		
*R*[*F* ^2^ > 2σ(*F* ^2^)], *wR*(*F* ^2^), *S*	0.048, 0.145, 1.12	0.034, 0.100, 1.09
No. of reflections	2139	1830
No. of parameters	175	177
No. of restraints	19	2
H-atom treatment	H atoms treated by a mixture of independent and constrained refinement	H atoms treated by a mixture of independent and constrained refinement
Δρ_max_, Δρ_min_ (e Å^−3^)	0.45, −0.43	0.30, −0.35
Absolute structure	–	Flack (1983 ▸), 973 Friedel pairs
Absolute structure parameter	–	0.08 (2)

Computer programs: *APEX2*, *SAINT-Plus* and *XPREP* (Bruker, 2009 ▸), *SHELXS97* and *SHELXL97* (Sheldrick, 2008 ▸) and *Mercury* (Macrae *et al.*, 2008 ▸).

## supplementary crystallographic information

### (I) 4-Methoxy-N-(4-methylphenyl)benzenesulfonamide Crystal data

**Table:**

C_14_H_15_NO_3_S	Prism
*M**_r_* = 277.33	*D*_x_ = 1.375 Mg m^−^^3^
Monoclinic, *P*2_1_/*c*	Cu *K*α radiation, λ = 1.54178 Å
*a* = 14.5604 (5) Å	Cell parameters from 178 reflections
*b* = 5.2459 (2) Å	θ = 5.0–64.1°
*c* = 17.6094 (6) Å	µ = 2.19 mm^−^^1^
β = 95.205 (2)°	*T* = 296 K
*V* = 1339.50 (8) Å^3^	Prism, colourless
*Z* = 4	0.33 × 0.27 × 0.21 mm
*F*(000) = 584	

### (I) 4-Methoxy-N-(4-methylphenyl)benzenesulfonamide Data collection

**Table:**

Bruker APEXII diffractometer	2047 reflections with *I* > 2σ(*I*)
Radiation source: fine-focus sealed tube	*R*_int_ = 0.042
Graphite monochromator	θ_max_ = 64.1°, θ_min_ = 5.0°
phi and φ scans	*h* = −16→16
Absorption correction: multi-scan (SADABS; Bruker, 2009)	*k* = −5→6
*T*_min_ = 0.517, *T*_max_ = 0.632	*l* = −20→20
7071 measured reflections	1 standard reflections every 1 reflections
2139 independent reflections	intensity decay: 0.1%

### (I) 4-Methoxy-N-(4-methylphenyl)benzenesulfonamide Refinement

**Table:**

Refinement on *F*^2^	Primary atom site location: structure-invariant direct methods
Least-squares matrix: full	Secondary atom site location: difference Fourier map
*R*[*F*^2^ > 2σ(*F*^2^)] = 0.048	Hydrogen site location: inferred from neighbouring sites
*wR*(*F*^2^) = 0.145	H atoms treated by a mixture of independent and constrained refinement
*S* = 1.12	*w* = 1/[σ^2^(*F*_o_^2^) + (0.095*P*)^2^ + 0.6515*P*] where *P* = (*F*_o_^2^ + 2*F*_c_^2^)/3
2139 reflections	(Δ/σ)_max_ = 0.001
175 parameters	Δρ_max_ = 0.45 e Å^−^^3^
19 restraints	Δρ_min_ = −0.43 e Å^−^^3^

### (I) 4-Methoxy-N-(4-methylphenyl)benzenesulfonamide Special details

**Table:**

Geometry. All s.u.'s (except the s.u. in the dihedral angle between two l.s. planes) are estimated using the full covariance matrix. The cell s.u.'s are taken into account individually in the estimation of s.u.'s in distances, angles and torsion angles; correlations between s.u.'s in cell parameters are only used when they are defined by crystal symmetry. An approximate (isotropic) treatment of cell s.u.'s is used for estimating s.u.'s involving l.s. planes.
Refinement. Refinement of *F*^2^ against ALL reflections. The weighted *R*-factor *wR* and goodness of fit *S* are based on *F*^2^, conventional *R*-factors *R* are based on *F*, with *F* set to zero for negative *F*^2^. The threshold expression of *F*^2^ > 2σ(*F*^2^) is used only for calculating *R*-factors(gt) *etc*. and is not relevant to the choice of reflections for refinement. *R*-factors based on *F*^2^ are statistically about twice as large as those based on *F*, and *R*- factors based on ALL data will be even larger.

### (I) 4-Methoxy-N-(4-methylphenyl)benzenesulfonamide Fractional atomic coordinates and isotropic or equivalent isotropic displacement parameters (Å^2^)

**Table:**

	*x*	*y*	*z*	*U*_iso_*/*U*_eq_	Occ. (<1)
H1	0.7020 (19)	0.376 (2)	0.4174 (15)	0.033 (7)*	
C1	0.87525 (15)	0.0577 (4)	0.41335 (12)	0.0223 (5)	
C2A	0.9220 (3)	0.2925 (9)	0.4259 (2)	0.0227 (12)*	0.516 (7)
H2A	0.8960	0.4215	0.4532	0.027*	0.516 (7)
C3A	1.0070 (3)	0.3298 (9)	0.3973 (3)	0.0243 (12)*	0.516 (7)
H3A	1.0392	0.4819	0.4057	0.029*	0.516 (7)
C2B	0.9452 (3)	0.1901 (9)	0.4561 (3)	0.0230 (13)*	0.484 (7)
H2B	0.9355	0.2534	0.5040	0.028*	0.484 (7)
C3B	1.0299 (3)	0.2268 (10)	0.4262 (3)	0.0231 (13)*	0.484 (7)
H3B	1.0768	0.3160	0.4540	0.028*	0.484 (7)
C4	1.04337 (15)	0.1285 (4)	0.35455 (12)	0.0226 (5)	
C5A	0.9913 (3)	−0.0795 (10)	0.3344 (3)	0.0190 (14)*	0.516 (7)
H5A	1.0125	−0.1992	0.3011	0.023*	0.516 (7)
C6A	0.9068 (4)	−0.1143 (11)	0.3631 (3)	0.0199 (16)*	0.516 (7)
H6A	0.8710	−0.2557	0.3483	0.024*	0.516 (7)
C5B	0.9778 (4)	−0.0372 (12)	0.3192 (4)	0.0215 (16)*	0.484 (7)
H5B	0.9903	−0.1226	0.2750	0.026*	0.484 (7)
C6B	0.8942 (4)	−0.0756 (12)	0.3492 (3)	0.0191 (17)*	0.484 (7)
H6B	0.8514	−0.1900	0.3264	0.023*	0.484 (7)
S1	0.76759 (3)	0.02277 (10)	0.45066 (3)	0.0195 (3)	
O3	1.12308 (11)	0.1523 (3)	0.32071 (9)	0.0257 (4)	
O1	0.77618 (12)	0.1184 (3)	0.52696 (8)	0.0288 (4)	
O2	0.73662 (11)	−0.2332 (3)	0.43553 (9)	0.0239 (4)	
N1	0.69436 (13)	0.2136 (3)	0.40319 (10)	0.0209 (5)	
C10	0.60286 (15)	0.1132 (4)	0.16969 (13)	0.0233 (5)	
C7	0.66683 (14)	0.1726 (4)	0.32369 (12)	0.0190 (5)	
C11	0.66087 (17)	0.3146 (5)	0.19337 (13)	0.0269 (5)	
H11	0.6785	0.4311	0.1576	0.032*	
C9	0.57863 (15)	−0.0581 (5)	0.22474 (14)	0.0251 (5)	
H9	0.5409	−0.1957	0.2099	0.030*	
C14	1.18279 (18)	0.3601 (5)	0.34384 (17)	0.0401 (7)	
H14A	1.1496	0.5177	0.3367	0.060*	
H14B	1.2345	0.3610	0.3137	0.060*	
H14C	1.2043	0.3410	0.3967	0.060*	
C8	0.60898 (15)	−0.0295 (4)	0.30080 (13)	0.0219 (5)	
H8	0.5908	−0.1449	0.3366	0.026*	
C12	0.69271 (16)	0.3439 (4)	0.26919 (12)	0.0236 (5)	
H12	0.7317	0.4791	0.2839	0.028*	
C13	0.56880 (18)	0.0780 (6)	0.08674 (14)	0.0363 (6)	
H13A	0.6139	−0.0147	0.0614	0.054*	
H13B	0.5589	0.2417	0.0631	0.054*	
H13C	0.5119	−0.0155	0.0831	0.054*	

### (I) 4-Methoxy-N-(4-methylphenyl)benzenesulfonamide Atomic displacement parameters (Å^2^)

**Table:**

	*U*^11^	*U*^22^	*U*^33^	*U*^12^	*U*^13^	*U*^23^
C1	0.0253 (12)	0.0218 (12)	0.0201 (11)	−0.0001 (10)	0.0032 (9)	−0.0043 (9)
C4	0.0233 (11)	0.0225 (12)	0.0222 (11)	0.0006 (9)	0.0021 (9)	0.0015 (9)
S1	0.0239 (4)	0.0169 (4)	0.0181 (4)	−0.0004 (2)	0.0046 (2)	−0.00107 (18)
O3	0.0223 (8)	0.0264 (9)	0.0290 (8)	−0.0022 (7)	0.0056 (7)	0.0003 (7)
O1	0.0336 (9)	0.0348 (10)	0.0187 (8)	0.0010 (8)	0.0062 (7)	−0.0046 (7)
O2	0.0294 (9)	0.0151 (8)	0.0277 (8)	−0.0003 (7)	0.0046 (7)	0.0038 (6)
N1	0.0286 (10)	0.0129 (10)	0.0220 (10)	0.0015 (8)	0.0061 (8)	−0.0028 (7)
C10	0.0199 (11)	0.0249 (13)	0.0254 (12)	0.0060 (9)	0.0027 (9)	−0.0035 (9)
C7	0.0186 (11)	0.0154 (11)	0.0236 (11)	0.0038 (8)	0.0046 (9)	−0.0017 (8)
C11	0.0322 (13)	0.0231 (12)	0.0258 (12)	0.0017 (10)	0.0060 (10)	0.0025 (9)
C9	0.0187 (11)	0.0223 (12)	0.0339 (13)	−0.0017 (10)	0.0008 (9)	−0.0041 (10)
C14	0.0262 (13)	0.0385 (15)	0.0570 (18)	−0.0111 (12)	0.0118 (12)	−0.0074 (13)
C8	0.0177 (11)	0.0179 (11)	0.0305 (13)	−0.0004 (9)	0.0042 (9)	0.0032 (9)
C12	0.0294 (12)	0.0145 (11)	0.0274 (12)	−0.0036 (9)	0.0051 (9)	−0.0021 (9)
C13	0.0329 (14)	0.0476 (17)	0.0281 (13)	0.0003 (13)	0.0019 (11)	−0.0059 (12)

### (I) 4-Methoxy-N-(4-methylphenyl)benzenesulfonamide Geometric parameters (Å, º)

**Table:**

C1—C6A	1.371 (5)	S1—O2	1.4340 (17)
C1—C6B	1.378 (5)	S1—N1	1.6360 (19)
C1—C2B	1.395 (5)	O3—C14	1.430 (3)
C1—C2A	1.415 (5)	N1—C7	1.437 (3)
C1—S1	1.763 (2)	N1—H1	0.893 (10)
C2A—C3A	1.391 (5)	C10—C9	1.391 (3)
C2A—H2A	0.9300	C10—C11	1.393 (3)
C3A—C4	1.426 (5)	C10—C13	1.511 (3)
C3A—H3A	0.9300	C7—C8	1.391 (3)
C2B—C3B	1.397 (6)	C7—C12	1.392 (3)
C2B—H2B	0.9300	C11—C12	1.382 (3)
C3B—C4	1.394 (5)	C11—H11	0.9300
C3B—H3B	0.9300	C9—C8	1.380 (3)
C4—C5A	1.358 (5)	C9—H9	0.9300
C4—O3	1.358 (3)	C14—H14A	0.9600
C4—C5B	1.395 (5)	C14—H14B	0.9600
C5A—C6A	1.385 (6)	C14—H14C	0.9600
C5A—H5A	0.9300	C8—H8	0.9300
C6A—H6A	0.9300	C12—H12	0.9300
C5B—C6B	1.385 (6)	C13—H13A	0.9600
C5B—H5B	0.9300	C13—H13B	0.9600
C6B—H6B	0.9300	C13—H13C	0.9600
S1—O1	1.4291 (16)		
			
C6A—C1—C2B	113.9 (3)	O1—S1—O2	120.18 (10)
C6B—C1—C2B	120.3 (3)	O1—S1—N1	105.25 (10)
C6A—C1—C2A	119.3 (3)	O2—S1—N1	107.39 (9)
C6B—C1—C2A	116.1 (3)	O1—S1—C1	107.99 (10)
C6A—C1—S1	122.2 (3)	O2—S1—C1	107.66 (10)
C6B—C1—S1	120.2 (3)	N1—S1—C1	107.83 (10)
C2B—C1—S1	118.7 (2)	C4—O3—C14	117.83 (18)
C2A—C1—S1	117.6 (2)	C7—N1—S1	121.15 (14)
C3A—C2A—C1	119.8 (4)	C7—N1—H1	115.7 (17)
C3A—C2A—H2A	120.1	S1—N1—H1	112.5 (18)
C1—C2A—H2A	120.1	C9—C10—C11	117.8 (2)
C2A—C3A—C4	118.1 (4)	C9—C10—C13	120.9 (2)
C2A—C3A—H3A	120.9	C11—C10—C13	121.3 (2)
C4—C3A—H3A	120.9	C8—C7—C12	119.2 (2)
C1—C2B—C3B	119.5 (4)	C8—C7—N1	120.31 (19)
C1—C2B—H2B	120.3	C12—C7—N1	120.4 (2)
C3B—C2B—H2B	120.3	C12—C11—C10	121.0 (2)
C4—C3B—C2B	119.4 (4)	C12—C11—H11	119.5
C4—C3B—H3B	120.3	C10—C11—H11	119.5
C2B—C3B—H3B	120.3	C8—C9—C10	121.8 (2)
C5A—C4—O3	116.0 (3)	C8—C9—H9	119.1
C5A—C4—C3B	114.4 (3)	C10—C9—H9	119.1
O3—C4—C3B	124.1 (3)	O3—C14—H14A	109.5
O3—C4—C5B	116.0 (3)	O3—C14—H14B	109.5
C3B—C4—C5B	119.2 (3)	H14A—C14—H14B	109.5
C5A—C4—C3A	120.5 (3)	O3—C14—H14C	109.5
O3—C4—C3A	122.4 (2)	H14A—C14—H14C	109.5
C5B—C4—C3A	115.1 (3)	H14B—C14—H14C	109.5
C4—C5A—C6A	120.3 (4)	C9—C8—C7	119.8 (2)
C4—C5A—H5A	119.9	C9—C8—H8	120.1
C6A—C5A—H5A	119.9	C7—C8—H8	120.1
C1—C6A—C5A	120.6 (4)	C11—C12—C7	120.4 (2)
C1—C6A—H6A	119.7	C11—C12—H12	119.8
C5A—C6A—H6A	119.7	C7—C12—H12	119.8
C6B—C5B—C4	120.7 (5)	C10—C13—H13A	109.5
C6B—C5B—H5B	119.7	C10—C13—H13B	109.5
C4—C5B—H5B	119.7	H13A—C13—H13B	109.5
C1—C6B—C5B	119.2 (5)	C10—C13—H13C	109.5
C1—C6B—H6B	120.4	H13A—C13—H13C	109.5
C5B—C6B—H6B	120.4	H13B—C13—H13C	109.5
			
C6A—C1—C2A—C3A	−10.4 (6)	S1—C1—C6B—C5B	177.4 (5)
C6B—C1—C2A—C3A	−26.8 (6)	C4—C5B—C6B—C1	1.9 (9)
C2B—C1—C2A—C3A	79.3 (5)	C6A—C1—S1—O1	145.9 (4)
S1—C1—C2A—C3A	−179.7 (3)	C6B—C1—S1—O1	163.2 (4)
C1—C2A—C3A—C4	1.2 (6)	C2B—C1—S1—O1	−7.0 (3)
C6A—C1—C2B—C3B	27.0 (6)	C2A—C1—S1—O1	−45.0 (3)
C6B—C1—C2B—C3B	11.9 (6)	C6A—C1—S1—O2	14.8 (4)
C2A—C1—C2B—C3B	−80.4 (5)	C6B—C1—S1—O2	32.1 (4)
S1—C1—C2B—C3B	−177.9 (3)	C2B—C1—S1—O2	−138.2 (3)
C1—C2B—C3B—C4	−0.6 (6)	C2A—C1—S1—O2	−176.2 (3)
C2B—C3B—C4—C5A	−27.1 (6)	C6A—C1—S1—N1	−100.8 (4)
C2B—C3B—C4—O3	−179.6 (3)	C6B—C1—S1—N1	−83.5 (4)
C2B—C3B—C4—C5B	−9.8 (6)	C2B—C1—S1—N1	106.3 (3)
C2B—C3B—C4—C3A	82.0 (6)	C2A—C1—S1—N1	68.2 (3)
C2A—C3A—C4—C5A	8.2 (6)	C5A—C4—O3—C14	−176.2 (4)
C2A—C3A—C4—O3	176.2 (3)	C3B—C4—O3—C14	−24.1 (4)
C2A—C3A—C4—C3B	−80.1 (6)	C5B—C4—O3—C14	165.9 (4)
C2A—C3A—C4—C5B	25.4 (6)	C3A—C4—O3—C14	15.3 (4)
O3—C4—C5A—C6A	−177.0 (4)	O1—S1—N1—C7	−178.58 (16)
C3B—C4—C5A—C6A	28.1 (7)	O2—S1—N1—C7	−49.43 (18)
C5B—C4—C5A—C6A	−82.9 (14)	C1—S1—N1—C7	66.33 (19)
C3A—C4—C5A—C6A	−8.3 (7)	S1—N1—C7—C8	71.9 (2)
C6B—C1—C6A—C5A	92.3 (16)	S1—N1—C7—C12	−112.4 (2)
C2B—C1—C6A—C5A	−26.5 (7)	C9—C10—C11—C12	−0.5 (3)
C2A—C1—C6A—C5A	10.5 (7)	C13—C10—C11—C12	−179.2 (2)
S1—C1—C6A—C5A	179.3 (4)	C11—C10—C9—C8	1.3 (3)
C4—C5A—C6A—C1	−1.2 (8)	C13—C10—C9—C8	180.0 (2)
C5A—C4—C5B—C6B	86.0 (14)	C10—C9—C8—C7	−1.2 (3)
O3—C4—C5B—C6B	179.8 (5)	C12—C7—C8—C9	0.4 (3)
C3B—C4—C5B—C6B	9.2 (8)	N1—C7—C8—C9	176.2 (2)
C3A—C4—C5B—C6B	−27.4 (7)	C10—C11—C12—C7	−0.3 (3)
C6A—C1—C6B—C5B	−80.7 (16)	C8—C7—C12—C11	0.4 (3)
C2B—C1—C6B—C5B	−12.5 (8)	N1—C7—C12—C11	−175.4 (2)
C2A—C1—C6B—C5B	25.2 (8)		

### (I) 4-Methoxy-N-(4-methylphenyl)benzenesulfonamide Hydrogen-bond geometry (Å, º)

Cg is the centroid of the C7–C12 ring.

**Table:**

*D*—H···*A*	*D*—H	H···*A*	*D*···*A*	*D*—H···*A*
N1—H1···O2^i^	0.89 (1)	2.13 (1)	3.010 (2)	170 (2)
C14—H14*B*···*Cg*^ii^	0.96	2.70	3.541 (2)	146
C9—H9···*Cg*^iii^	0.93	2.87	3.560 (2)	132

Symmetry codes: (i) *x*, *y*+1, *z*; (ii) −*x*, *y*+1/2, −*z*+1/2; (iii) −*x*+1, *y*−1/2, −*z*+1/2.

### (II) N-(4-Fluorophenyl)-4-methoxybenzenesulfonamide Crystal data

**Table:**

C_13_H_12_FNO_3_S	Prism
*M**_r_* = 281.30	*D*_x_ = 1.473 Mg m^−^^3^
Orthorhombic, *P**n**a*2_1_	Cu *K*α radiation, λ = 1.54178 Å
Hall symbol: P 2c -2n	Cell parameters from 143 reflections
*a* = 20.2188 (13) Å	θ = 4.3–64.1°
*b* = 12.1199 (8) Å	µ = 2.44 mm^−^^1^
*c* = 5.1770 (3) Å	*T* = 296 K
*V* = 1268.62 (14) Å^3^	Prism, colourless
*Z* = 4	0.32 × 0.27 × 0.22 mm
*F*(000) = 584	

### (II) N-(4-Fluorophenyl)-4-methoxybenzenesulfonamide Data collection

**Table:**

Bruker APEXII diffractometer	1784 reflections with *I* > 2σ(*I*)
Radiation source: fine-focus sealed tube	*R*_int_ = 0.037
Graphite monochromator	θ_max_ = 64.1°, θ_min_ = 4.3°
phi and φ scans	*h* = −22→23
Absorption correction: multi-scan (SADABS; Bruker, 2009)	*k* = −13→14
*T*_min_ = 0.481, *T*_max_ = 0.585	*l* = −5→5
5438 measured reflections	1 standard reflections every 1 reflections
1830 independent reflections	intensity decay: 0.1%

### (II) N-(4-Fluorophenyl)-4-methoxybenzenesulfonamide Refinement

**Table:**

Refinement on *F*^2^	Secondary atom site location: difference Fourier map
Least-squares matrix: full	Hydrogen site location: inferred from neighbouring sites
*R*[*F*^2^ > 2σ(*F*^2^)] = 0.034	H atoms treated by a mixture of independent and constrained refinement
*wR*(*F*^2^) = 0.100	*w* = 1/[σ^2^(*F*_o_^2^) + (0.0719*P*)^2^] where *P* = (*F*_o_^2^ + 2*F*_c_^2^)/3
*S* = 1.09	(Δ/σ)_max_ = 0.001
1830 reflections	Δρ_max_ = 0.30 e Å^−^^3^
177 parameters	Δρ_min_ = −0.35 e Å^−^^3^
2 restraints	Absolute structure: Flack (1983), 973 Friedel pairs
Primary atom site location: structure-invariant direct methods	Absolute structure parameter: 0.08 (2)

### (II) N-(4-Fluorophenyl)-4-methoxybenzenesulfonamide Special details

**Table:**

Geometry. All s.u.'s (except the s.u. in the dihedral angle between two l.s. planes) are estimated using the full covariance matrix. The cell s.u.'s are taken into account individually in the estimation of s.u.'s in distances, angles and torsion angles; correlations between s.u.'s in cell parameters are only used when they are defined by crystal symmetry. An approximate (isotropic) treatment of cell s.u.'s is used for estimating s.u.'s involving l.s. planes.
Refinement. Refinement of *F*^2^ against ALL reflections. The weighted *R*-factor *wR* and goodness of fit *S* are based on *F*^2^, conventional *R*-factors *R* are based on *F*, with *F* set to zero for negative *F*^2^. The threshold expression of *F*^2^ > 2σ(*F*^2^) is used only for calculating *R*-factors(gt) *etc*. and is not relevant to the choice of reflections for refinement. *R*-factors based on *F*^2^ are statistically about twice as large as those based on *F*, and *R*- factors based on ALL data will be even larger.

### (II) N-(4-Fluorophenyl)-4-methoxybenzenesulfonamide Fractional atomic coordinates and isotropic or equivalent isotropic displacement parameters (Å^2^)

**Table:**

	*x*	*y*	*z*	*U*_iso_*/*U*_eq_	
S1	0.77164 (2)	0.42206 (4)	0.40154 (13)	0.0172 (2)	
F1	0.96703 (8)	−0.00461 (12)	0.4446 (3)	0.0328 (4)	
O1	0.75213 (8)	0.53031 (14)	0.4790 (4)	0.0229 (4)	
O2	0.79392 (9)	0.40209 (15)	0.1426 (4)	0.0234 (4)	
O3	0.56267 (8)	0.10112 (16)	0.6458 (4)	0.0289 (5)	
N1	0.83314 (9)	0.38833 (18)	0.5945 (4)	0.0179 (5)	
C10	0.93385 (12)	0.0915 (2)	0.4822 (6)	0.0245 (6)	
C6	0.70105 (11)	0.2317 (2)	0.3318 (5)	0.0224 (6)	
H6	0.7303	0.2162	0.1979	0.027*	
C8	0.85326 (11)	0.1947 (2)	0.7108 (5)	0.0229 (6)	
H8	0.8215	0.2003	0.8401	0.028*	
C7	0.86643 (11)	0.2844 (2)	0.5534 (5)	0.0181 (5)	
C4	0.60893 (10)	0.1812 (2)	0.5970 (6)	0.0216 (6)	
C2	0.66316 (11)	0.3540 (2)	0.6686 (6)	0.0221 (5)	
H2	0.6672	0.4199	0.7591	0.027*	
C5	0.65224 (10)	0.15819 (19)	0.3967 (6)	0.0234 (5)	
H5	0.6482	0.0924	0.3056	0.028*	
C11	0.94793 (12)	0.1792 (2)	0.3219 (6)	0.0277 (6)	
H11	0.9794	0.1731	0.1917	0.033*	
C12	0.91355 (11)	0.2773 (2)	0.3611 (5)	0.0240 (6)	
H12	0.9224	0.3382	0.2575	0.029*	
C3	0.61372 (11)	0.2795 (2)	0.7337 (5)	0.0234 (6)	
H3	0.5843	0.2952	0.8667	0.028*	
C1	0.70619 (12)	0.3303 (2)	0.4696 (5)	0.0186 (5)	
C13	0.51905 (13)	0.1183 (2)	0.8577 (6)	0.0330 (7)	
H13A	0.5443	0.1268	1.0134	0.049*	
H13B	0.4901	0.0560	0.8745	0.049*	
H13C	0.4933	0.1837	0.8281	0.049*	
C9	0.88759 (13)	0.0962 (2)	0.6755 (6)	0.0261 (6)	
H9	0.8794	0.0353	0.7801	0.031*	
H1	0.8245 (14)	0.399 (2)	0.763 (2)	0.023 (8)*	

### (II) N-(4-Fluorophenyl)-4-methoxybenzenesulfonamide Atomic displacement parameters (Å^2^)

**Table:**

	*U*^11^	*U*^22^	*U*^33^	*U*^12^	*U*^13^	*U*^23^
S1	0.0222 (3)	0.0161 (3)	0.0134 (3)	−0.0004 (2)	−0.0004 (2)	−0.0003 (2)
F1	0.0403 (8)	0.0248 (8)	0.0333 (10)	0.0127 (6)	0.0008 (8)	−0.0014 (8)
O1	0.0286 (8)	0.0160 (9)	0.0241 (10)	0.0003 (7)	−0.0029 (7)	−0.0009 (7)
O2	0.0280 (9)	0.0249 (10)	0.0172 (10)	−0.0017 (8)	0.0010 (8)	−0.0009 (8)
O3	0.0258 (9)	0.0260 (10)	0.0349 (13)	−0.0065 (8)	0.0066 (9)	−0.0040 (9)
N1	0.0228 (9)	0.0193 (11)	0.0117 (11)	−0.0006 (8)	0.0019 (9)	−0.0019 (9)
C10	0.0262 (12)	0.0222 (14)	0.0251 (15)	0.0056 (11)	−0.0031 (11)	−0.0029 (11)
C6	0.0239 (11)	0.0208 (13)	0.0225 (15)	0.0030 (10)	0.0038 (10)	−0.0064 (11)
C8	0.0221 (10)	0.0265 (15)	0.0202 (14)	0.0010 (10)	0.0050 (10)	0.0016 (11)
C7	0.0202 (11)	0.0186 (13)	0.0155 (13)	−0.0019 (10)	−0.0016 (9)	−0.0015 (10)
C4	0.0201 (11)	0.0181 (13)	0.0265 (14)	−0.0009 (9)	−0.0039 (11)	0.0023 (11)
C2	0.0268 (12)	0.0202 (13)	0.0193 (13)	−0.0003 (10)	−0.0019 (10)	−0.0052 (11)
C5	0.0258 (11)	0.0183 (12)	0.0261 (14)	−0.0003 (9)	−0.0027 (11)	−0.0081 (13)
C11	0.0279 (11)	0.0306 (15)	0.0246 (17)	0.0054 (12)	0.0065 (11)	−0.0005 (12)
C12	0.0275 (11)	0.0227 (12)	0.0218 (14)	0.0003 (10)	0.0065 (11)	0.0023 (11)
C3	0.0234 (11)	0.0268 (14)	0.0201 (14)	−0.0002 (11)	0.0034 (10)	−0.0032 (11)
C1	0.0212 (11)	0.0182 (12)	0.0162 (12)	0.0018 (10)	−0.0026 (9)	−0.0010 (10)
C13	0.0272 (12)	0.0401 (15)	0.0316 (17)	−0.0092 (12)	0.0027 (12)	0.0013 (14)
C9	0.0316 (13)	0.0208 (14)	0.0260 (16)	−0.0008 (11)	−0.0004 (12)	0.0050 (12)

### (II) N-(4-Fluorophenyl)-4-methoxybenzenesulfonamide Geometric parameters (Å, º)

**Table:**

S1—O1	1.4275 (18)	C8—H8	0.9300
S1—O2	1.435 (2)	C7—C12	1.380 (3)
S1—N1	1.647 (2)	C4—C5	1.385 (4)
S1—C1	1.764 (2)	C4—C3	1.390 (4)
F1—C10	1.358 (3)	C2—C1	1.378 (4)
O3—C4	1.371 (3)	C2—C3	1.388 (4)
O3—C13	1.423 (3)	C2—H2	0.9300
N1—C7	1.444 (3)	C5—H5	0.9300
N1—H1	0.898 (10)	C11—C12	1.392 (4)
C10—C9	1.371 (4)	C11—H11	0.9300
C10—C11	1.378 (4)	C12—H12	0.9300
C6—C5	1.372 (3)	C3—H3	0.9300
C6—C1	1.395 (3)	C13—H13A	0.9600
C6—H6	0.9300	C13—H13B	0.9600
C8—C7	1.385 (4)	C13—H13C	0.9600
C8—C9	1.394 (4)	C9—H9	0.9300
			
O1—S1—O2	120.28 (11)	C1—C2—H2	120.0
O1—S1—N1	105.44 (11)	C3—C2—H2	120.0
O2—S1—N1	106.74 (11)	C6—C5—C4	120.5 (2)
O1—S1—C1	108.44 (11)	C6—C5—H5	119.8
O2—S1—C1	108.41 (11)	C4—C5—H5	119.8
N1—S1—C1	106.77 (11)	C10—C11—C12	117.9 (2)
C4—O3—C13	117.5 (2)	C10—C11—H11	121.1
C7—N1—S1	118.62 (16)	C12—C11—H11	121.1
C7—N1—H1	111.3 (19)	C7—C12—C11	120.2 (2)
S1—N1—H1	113.8 (19)	C7—C12—H12	119.9
F1—C10—C9	118.5 (2)	C11—C12—H12	119.9
F1—C10—C11	118.3 (2)	C2—C3—C4	118.9 (2)
C9—C10—C11	123.3 (2)	C2—C3—H3	120.5
C5—C6—C1	119.0 (2)	C4—C3—H3	120.5
C5—C6—H6	120.5	C2—C1—C6	120.9 (2)
C1—C6—H6	120.5	C2—C1—S1	119.40 (19)
C7—C8—C9	120.0 (2)	C6—C1—S1	119.56 (19)
C7—C8—H8	120.0	O3—C13—H13A	109.5
C9—C8—H8	120.0	O3—C13—H13B	109.5
C12—C7—C8	120.5 (2)	H13A—C13—H13B	109.5
C12—C7—N1	118.9 (2)	O3—C13—H13C	109.5
C8—C7—N1	120.5 (2)	H13A—C13—H13C	109.5
O3—C4—C5	115.2 (2)	H13B—C13—H13C	109.5
O3—C4—C3	124.1 (2)	C10—C9—C8	118.1 (3)
C5—C4—C3	120.7 (2)	C10—C9—H9	121.0
C1—C2—C3	120.0 (2)	C8—C9—H9	121.0
			
O1—S1—N1—C7	−176.37 (18)	C1—C2—C3—C4	0.5 (4)
O2—S1—N1—C7	−47.4 (2)	O3—C4—C3—C2	179.3 (2)
C1—S1—N1—C7	68.4 (2)	C5—C4—C3—C2	−0.8 (4)
C9—C8—C7—C12	−0.1 (4)	C3—C2—C1—C6	−0.2 (4)
C9—C8—C7—N1	−177.5 (2)	C3—C2—C1—S1	−176.4 (2)
S1—N1—C7—C12	80.1 (3)	C5—C6—C1—C2	0.1 (4)
S1—N1—C7—C8	−102.5 (2)	C5—C6—C1—S1	176.26 (19)
C13—O3—C4—C5	177.0 (2)	O1—S1—C1—C2	−27.8 (2)
C13—O3—C4—C3	−3.0 (4)	O2—S1—C1—C2	−159.9 (2)
C1—C6—C5—C4	−0.3 (4)	N1—S1—C1—C2	85.4 (2)
O3—C4—C5—C6	−179.4 (2)	O1—S1—C1—C6	156.0 (2)
C3—C4—C5—C6	0.7 (4)	O2—S1—C1—C6	23.8 (2)
F1—C10—C11—C12	179.8 (2)	N1—S1—C1—C6	−90.8 (2)
C9—C10—C11—C12	0.5 (4)	F1—C10—C9—C8	−179.3 (2)
C8—C7—C12—C11	0.6 (4)	C11—C10—C9—C8	0.0 (4)
N1—C7—C12—C11	178.0 (2)	C7—C8—C9—C10	−0.2 (4)
C10—C11—C12—C7	−0.8 (4)		

### (II) N-(4-Fluorophenyl)-4-methoxybenzenesulfonamide Hydrogen-bond geometry (Å, º)

**Table:**

*D*—H···*A*	*D*—H	H···*A*	*D*···*A*	*D*—H···*A*
N1—H1···O2^i^	0.90 (1)	2.06 (1)	2.951 (3)	171 (3)
C6—H6···O1^ii^	0.93	2.55	3.192 (3)	127
C13—H13*B*···O3^iii^	0.96	2.60	3.468 (3)	151

Symmetry codes: (i) *x*, *y*, *z*+1; (ii) −*x*+3/2, *y*−1/2, *z*−1/2; (iii) −*x*+1, −*y*, *z*+1/2.
